# Supplementary material for: Proteomic analysis of extracellular vesicles released from endothelial cells in vitro reveals increased levels of E-selectin and dual specificity phosphatase 7 as a potential marker of TNFα-mediated apoptosis
Source: PLoS One. 2026 Jun 26;21(6):e0352394. doi: 10.1371/journal.pone.0352394 (PMC13308812; doi:10.1371/journal.pone.0352394)
Supplement: S2 File — (PDF) [file pone.0352394.s002.pdf]

Supplemental Data File 2. LUT file

Bin number	*Collision Energy (v)*
1	0
2	0.502513
3	1.005025
4	1.507538
5	2.01005
6	2.512563
7	3.015075
8	3.517588
9	4.020101
10	4.522613
11	5.025126
12	5.527638
13	6.030151
14	6.532663
15	7.035176
16	7.537688
17	8.040201
18	8.542714
19	9.045226
20	9.547739
21	10.05025
22	10.55276
23	11.05528
24	11.55779
25	12.0603
26	12.56281
27	13.06533
28	13.56784
29	14.07035
30	14.57286
31	15.07538
32	15.57789
33	16.0804
34	16.58292
35	17.08543
36	17.58794
37	18.09045
38	18.59297
39	19.09548
40	19.59799
41	20.1005
42	20.60302
43	21.10553
44	21.60804
45	22.11055

46	22.61307
47	23.11558
48	23.61809
49	24.1206
50	24.62312
51	25.12563
52	25.62814
53	26.13065
54	26.63317
55	27.13568
56	27.63819
57	28.1407
58	28.64322
59	29.14573
60	29.64824
61	30.15075
62	30.65327
63	31.15578
64	31.65829
65	32.1608
66	32.66332
67	33.16583
68	33.66834
69	34.17085
70	34.67337
71	35.17588
72	35.67839
73	36.18091
74	36.68342
75	37.18593
76	37.68844
77	38.19096
78	38.69347
79	39.19598
80	39.69849
81	40.20101
82	40.70352
83	41.20603
84	41.70854
85	42.21106
86	42.71357
87	43.21608
88	43.71859
89	44.22111
90	44.72362
91	45.22613
92	45.72864
93	46.23116
94	46.73367
95	47.23618

96	47.73869
97	48.24121
98	48.74372
99	49.24623
100	49.74874
101	50.25126
102	50.75377
103	51.25628
104	51.75879
105	52.26131
106	52.76382
107	53.26633
108	53.76884
109	54.27136
110	54.77387
111	55.27638
112	55.77889
113	56.28141
114	56.78392
115	57.28643
116	57.78895
117	58.29146
118	58.79397
119	59.29648
120	59.799
121	60.30151
122	60.80402
123	61.30653
124	61.80905
125	62.31156
126	62.81407
127	63.31658
128	63.8191
129	64.32161
130	64.82412
131	65.32663
132	65.82915
133	66.33166
134	66.83417
135	67.33668
136	67.8392
137	68.34171
138	68.84422
139	69.34673
140	69.84925
141	70.35176
142	70.85427
143	71.35678
144	71.8593
145	72.36181

146	72.86432
147	73.36683
148	73.86935
149	74.37186
150	74.87437
151	75.37688
152	75.8794
153	76.38191
154	76.88442
155	77.38694
156	77.88945
157	78.39196
158	78.89447
159	79.39699
160	79.8995
161	80.40201
162	80.90452
163	81.40704
164	81.90955
165	82.41206
166	82.91457
167	83.41709
168	83.9196
169	84.42211
170	84.92462
171	85.42714
172	85.92965
173	86.43216
174	86.93467
175	87.43719
176	87.9397
177	88.44221
178	88.94472
179	89.44724
180	89.94975
181	90.45226
182	90.95477
183	91.45729
184	91.9598
185	92.46231
186	92.96482
187	93.46734
188	93.96985
189	94.47236
190	94.97487
191	95.47739
192	95.9799
193	96.48241
194	96.98493
195	97.48744

196	97.98995
197	98.49246
198	98.99498
199	99.49749
200	100
